# Analyzing Quantitative Trait Loci for Fiber Quality and Yield-Related Traits From a Recombinant Inbred Line Population With *Gossypium hirsutum* Race *palmeri* as One Parent

**DOI:** 10.3389/fpls.2021.817748

**Published:** 2022-01-03

**Authors:** Xueying Liu, Le Yang, Jinxia Wang, Yaqing Wang, Zhongni Guo, Qingqing Li, Jinming Yang, Youlin Wu, Li Chen, Zhonghua Teng, Dajun Liu, Dexin Liu, Kai Guo, Zhengsheng Zhang

**Affiliations:** College of Agronomy and Biotechnology, Southwest University, Chongqing, China

**Keywords:** genetic map, fiber quality, yield related traits, QTL, *Gossypium hirsutum* race *palmeri*

## Abstract

Fiber quality and yield-related traits are important agronomic traits in cotton breeding. To detect the genetic basis of fiber quality and yield related traits, a recombinant inbred line (RIL) population consisting of 182 lines was established from a cross between *Gossypium hirsutum* cultivar CCRI35 and *G. hirsutum* race *palmeri* accession TX-832. The RIL population was deeply genotyped using SLAF-seq and was phenotyped in six environments. A high-density genetic linkage map with 15,765 SNP markers and 153 SSR markers was constructed, with an average distance of 0.30 cM between adjacent markers. A total of 210 fiber quality quantitative trait loci (QTLs) and 73 yield-related QTLs were identified. Of the detected QTLs, 62 fiber quality QTLs and 10 yield-related QTLs were stable across multiple environments. Twelve and twenty QTL clusters were detected on the At and Dt subgenome, respectively. Twenty-three major QTL clusters were further validated through associated analysis and five candidate genes of four stable fiber quality QTLs were identified. This study revealed elite loci influencing fiber quality and yield and significant phenotypic selection regions during *G. hirsutum* domestication, and set a stage for future utilization of molecular marker assisted breeding in cotton breeding programs.

## Introduction

Cotton, with more than 7,000 years of cultivation history, is the most significant natural fiber crop and an important resource of plant protein and oil (Yuan et al., 2015; Wang M. et al., 2017). Besides textile fiber, cotton seeds could be processed into foodstuff, feed, and biofuel (Coppock et al., 1987; Liu et al., 2002, 2017). The economic impact of cotton is estimated to be approximately 500 billion dollars per year worldwide (Chen et al., 2007). With the rapid development of spinning industry and the increasing human demand, enhancing the fiber quality and yield has been a crucial goal of cotton breeding (Fang X. et al., 2017).

*Gossypium hirsutum* (upland cotton), which has experienced a long period of domestication for its high yield traits (Zhang et al., 2005; Hu et al., 2019), provides more than 90% of global cotton output. However, the genetic diversity of *G. hirsutum* cultivars are narrow and thus decreased the opportunities for identifying favorable alleles and breeding new cotton cultivars with more eminent yield and quality (Zhang et al., 2013; Okubazghi et al., 2017; Li et al., 2019). *G. hirsutum* races, also known as semiwild accessions, are primitive type of cultivated upland cotton (Hutchinson, 1951; Lacape et al., 2006; Feng et al., 2019). *G. hirsutum* races are classified into seven races (*yucatanense*, *marie-galante*, *morrlli*, *richmondii*, *punctatum*, *palmeri* and *latifolium*) according to their geographical distribution (Brubaker et al., 1999; Lacape et al., 2006). They were confirmed with promising phenotypes, including resistance to biotic stress and abiotic stress, and agronomically important traits such as variable fiber quality and yield traits (Okubazghi et al., 2017; Zhang et al., 2019). *G. hirsutum* races could be crossed with cultivars directly and therefore were gradually utilized to broaden the genetic basis of modern cotton cultivars and improve fiber quality and yield-related traits (Wang Y. et al., 2016; Okubazghi et al., 2017; Feng et al., 2019).

Yield-related and fiber quality traits are complex quantitative traits that were controlled by multiple genes and were affected by environments (Paterson et al., 2003; Said et al., 2013). The negative relation between fiber quality and yield traits limited the efficiency of conventional breeding methods (Yu et al., 2012; Zhang et al., 2014). A majority of previous quantitative trait locus (QTL) studies for fiber quality and yield-related traits were based on upland cotton intraspecific population and interspecific, whereas a few of them were conducted with *G. hirsutum* race accessions (Zhang et al., 2016; Feng et al., 2019). In this work, an accession TX-832 from *G. hirsutum* race *palmeri*, which is distributed in the Mexican States of Guerrero and Oaxaca and is characterized with distinctive laciniate leaves and small bolls, was utilized to detect elite loci influencing fiber quality and yield-related traits (Brubaker et al., 1999). A recombinant inbred line (RIL) population containing 182 lines from a cross between *G. hirsutum* cultivar CCRI35 and *G. hirsutum* race *palmeri* accession TX-832 were established. Single nucleotide polymorphism (SNP) markers obtained from Specific-Locus Amplified Fragment Sequencing (SLAF-seq) were applied to construct a high-density genetic linkage map. Polymorphic simple sequence repeats (SSR) markers were utilized to verify the accuracy of SNP markers and enrich the genetic map. Environment-stable QTLs and QTL clusters for fiber quality and yield-related traits were detected based on this genetic map. Major QTL clusters were further validated by SNP associated analysis, and five candidate genes related to fiber quality were identified. This work provided elite loci influencing fiber quality, yield-related traits, and valuable insights into the phenotypic selection regions during *G. hirsutum* domestication. The results built the basis for breeding cotton cultivars with high fiber quality and yield.

## Materials and Methods

### Mapping Population

The *G. hirsutum* cultivar CCRI35 and the *G. hirsutum* race *palmeri* accession TX-832 were crossed at Southwest University, Chongqing, China, in the summer of 2016. CCRI35 has fine performance in yield and was widely planted in China in the 2000s. TX-832, which was provided by Chinese cotton research academy, is characterized with features such as okra leaf and small boll. The F_1_ seeds were planted at Hainan, China, in the winter of 2016. One hundred and eighty-two F_2_ individuals were randomly selected at Chongqing in the subsequent summer, and then single-seed descent was conducted from the F_2:3_ generation to the F_2:6_ generation to complete a RIL population in the summer of 2019. The RIL population, as well as the two parents, were planted in totally six environments in China, including Beibei in Chongqing province (19CQ), Changsha in Hunan province (19CS), Sanya in Hainan province (19SY), and Kuitun in Xinjiang province (19KT) in 2019, Kuitun (20KT) and Kuerle (20KL) in Xinjiang province in 2020.

### Phenotyping and Analysis

All the mature bolls of the 182 lines and the two parents were hand-harvested in each environment. Their fiber samples were evaluated for five fiber quality traits, including fiber length (FL, mm), fiber micronaire (FM), fiber uniformity ratio (FU,%), fiber strength (FS, cN/tex), and fiber elongation (FE,%), using the high volume instrument (HIV) system at the Supervision Inspection and Testing Cotton Quality Center, Anyang, China. Three yield-related traits, including boll weight (BW, g), seed index (g), and lint percentage (LP,%) were collected from all environments. Besides, the yield-related traits lint index (LI, g) was also tested in the three environments. Microsoft Excel 2019 was applied to analysis the variance of each trait. SPSS 20.0 was used to analyze the correlations among traits. The R package “ggplot2” was applied to draw the visualized plots of traits performance and correlations in each environment.

### DNA Extraction and Specific-Locus Amplified Fragment Library Construction

Total genomic DNA of the two parents and the RIL population were extracted using modified CTAB method as described by Zhang et al. (2005). For SLAF-Seq library construction, two endonucleases *Hae*III and Hpy166II were finally chosen, based on the *in silico* analysis of *G. hirsutum* reference genome (TM-1, V2.1) (Hu et al., 2019), to digest the genomic DNA. SLAF labels with length of 414–464 bp were chosen for library construction and sequencing. The procedures of quality control, reads filter and trim, reads mapping and SNPs detection were carried out, as described by Wang et al. (2019).

### Marker Filtration and Map Construction

Single nucleotide polymorphisms in each SLAF locus were filtered as described by Wang et al. (2019). SSR markers were obtained previously in our laboratory by polymorphism screening between the two parents. The genetic map was constructed by Joinmap 4.0 using both SLAF markers and SSR markers (Van Ooijen, 2006). Chip-squared tests were applied to detected markers that deviated significantly from the expected Mendelian 1:1 segregation ratio with their *p*-value < 0.05. Regions that contained more than three adjacent significant segregation distorted loci were determined as segregation distortion region (SDR) (Liu et al., 2017).

### Quantitative Trait Loci Identification

Identification of QTLs and evaluation of their effects were carried out by Interval Mapping methods in MapQTL 6.0 (Van Ooijen, 2009). The threshold of odds ratio (LOD) to determine QTL was set to 3.0. QTLs identified in at least two (LI) or at least three (BW, SI, LP, FL, FS, FM, FU, and FE) environments were considered to be stable QTLs. A region contained more than three QTLs was determined as QTL cluster, and clusters that contained QTLs with their PVE value larger than 10% and LOD value larger than 5.0 were determined as major QTL clusters.

### Candidate Gene Identification

The physical regions of QTLs within the *G. hirsutum* reference genome (TM-1, V2.1) were determined by physical locations of flanking markers of their genetical confidence interval.

Transcriptome sequencing data from *G. hirsutum* TM-1 ovules including 0 days post-anthesis (DPA) and 5 DPA, and fibers at 10 DPA, 15 DPA and 25 DPA were acquired from the Cotton Omics Database^1^.

DNA libraries for whole genome re-sequencing were created for the CCRI35 and TX-832 and sequenced in an Illumina HiSeq platform. The procedures of reads mapping and SNP/Indel calling were carried out as described by Liu et al. (2020). Variations annotation was performed according the *G. hirsutum* reference genome (TM-1, V2.1) with the ANNOVAR package (Wang et al., 2010).

### Associated Analysis

The association analysis was conduct with public data released by Ma Z. et al. (2018). SNP Markers in major QTL clusters were firstly converted to their corresponding position of the reference genome (TM-1, V1.1) (Zhang et al., 2015). Genotype of common SNPs (SNPs sharing the same genome position with the present study) and phenotype data of 419 *G. hirsutum* accessions from study of Ma Z. et al. (2018) were analyzed. The software PLINK 2.0 (Chang et al., 2015) was applied to carry out association analysis.

## Results

### Phenotypic Variation and Correlation of Traits

**FIGURE 1:**
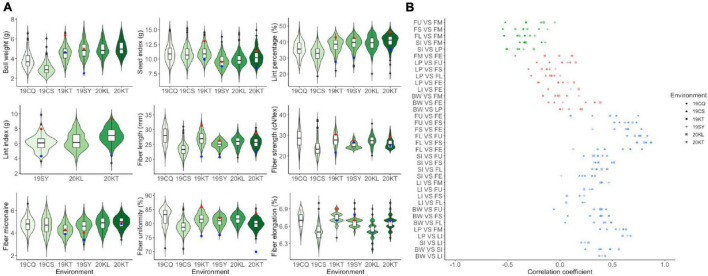
The phenotype performance **(A)** and the correlation analysis **(B)** among fiber quality and yield-related traits. Red and blue point in figure a suggesting the trait performance of CCRI35 and TX-832, respectively.

The descriptive statistics of all traits for the two parents and the RIL population were summarized in Supplementary Table 1 and displayed in Figure 1A. CCRI35 performed more superiorly than TX-832 in all yield related traits and most fiber quality traits including fiber length, fiber strength and fiber uniformity. The fiber micronaire performance of TX-832 was better than CCRI35, whereas the fiber elongation traits were comparable between the two parents. All the traits were continuous and approximately normal distributed, indicating that they are typical quantitative traits. Furthermore, transgressive segregation was observed for all fiber quality and yield related traits.

All the four yield related traits and the five fiber quality traits varied with environments (Figure 1A). The result of ANOVA also showed that all traits were significantly affected by both genetic and environmental factors (Supplementary Table 2).

Correlation analysis between traits pairs across all the environments was carried out separately (Supplementary Table 3). Three categories of correlativity were observed including significantly positive related, significantly negatively related and weakly related (Figure 1B). Among them, BW, SI, FL, FS were positively correlated with most traits except for LP and FM. FM was significantly positive correlated with LP and LI, whereas was negatively correlated with the other traits. LI was almost positively correlated with all the other traits except for FE.

### Analysis of Specific-Locus Amplified Fragment-Seq Data and Specific-Locus Amplified Fragment Labels

A total of 2,060.13 M paired-end reads (including 18.50 M reads for CCRI35, 166.97 M reads for TX-832 and the others for the RIL population) were generated from the SLAF library, with 95.50% of the bases were of high quality with Q30 and the average GC content was 37.81%. Totally, among the 400,313 SLAFs, 260,123 SLAFs located in the At subgenome and 129,614 SLAFs located in the Dt subgenome. The number of SLAFs of CCRI35 and TX-832 were 397,774 and 397,252, with average sequencing depth were 31.29-fold and 351.25-fold, respectively. For individuals in the RIL population, the SLAF numbers ranged from 302,115 to 398,465, the average sequencing depth ranged from 9.93-fold to 95.92-fold. Among the developed SLAFs on 26 chromosomes, 195,449 were polymorphic with a polymorphism rate of 50.15% (Supplementary Table 4 and Supplementary Figure 1). Chromosome A01, A06, A10 and D03 harbored the more than 60% polymorphic SLAFs, while the polymorphism rate of chromosome A04, with a number of 23.60%, was the least.

### Genotyping and Construction of the Genetic Map

**FIGURE 2:**
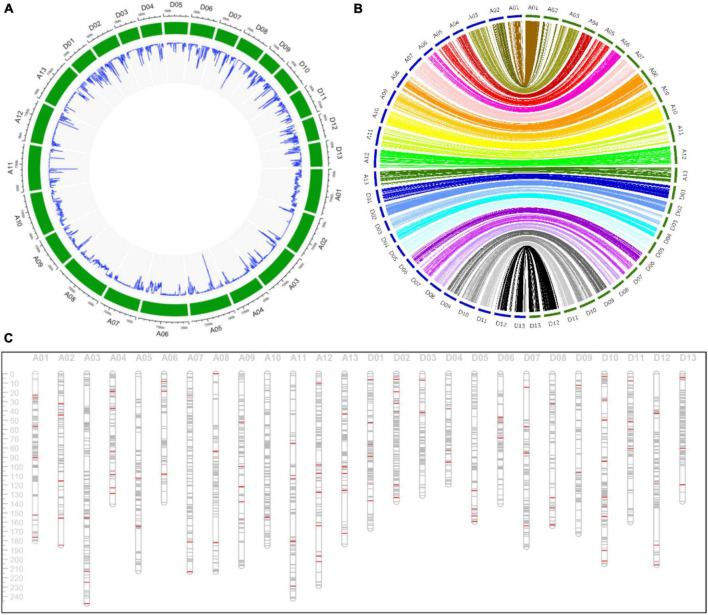
Detailed information about the genetic linkage map. **(A)** The density of SNP markers in the *G. hirsutum* genome in a 500 Kb window. **(B)** The collinearity analysis of markers between the genetic map and the physical map. The blue circle and the green circle indicating the genetic map and the physical map, respectively. **(C)** The distribution of markers in the genetic map. The gray lines and the red lines indicating SNP and SSR markers, respectively.

A total of 2,962,929 SNP markers were generated from these SLAFs, and 82,920 SNPs with genotype aa × bb were remained after filtering. These SNPs were further filtered with criteria including minimum read depth of parents less than 10, minimum read depth of offspring less than two and more than 25% missing data. Finally, the offspring lines were genotyped with aa, ab and bb using 15,765 filtered SNP markers to constructed the genetic linkage map. The density of these SNPs on the *G. hirsutum* genome was displayed in Figure 2A.

Simple sequence repeat primers were screened with polymorphism between CCRI35 and TX-832 to constructed a genetic linkage map using (CCRI35 × TX-832) F_2_ population previously in our laboratory. In this study, 153 polymorphic SSR markers distributed on all the 26 chromosomes were chosen to obtain the genotype of the RIL population and further verified the genotype of SNP markers. Names of these SSR markers were listed in Supplementary Table 5.

A high-density consensus genetic map including 15,765 SNP markers and 153 SSR markers were constructed, with a total length of 4,665.59 cM and an average distance of 0.30 cM between adjacent markers (Table 1). The At subgenome spanned 2,578.69 cM with 8,829 markers (8,750 SNP markers and 79 SSR markers), and the Dt subgenome spanned 2,086.90 cM with 7,015 SNP markers and 74 SSR markers. Chromosome A01, spanning 180.44 cM, contained the maximum markers including 1,327 SNP and 8 SSR markers. While chromosome D05 harbored only 94 SNP and 5 SSR markers with genetic length of 159.35 cM. Chromosome A03 and D04 was the longest and the shortest chromosome, respectively.

**TABLE 1:** Detailed information of the high-density genetic linkage map.

Chromosome	Length	No.	No.	Coverage	Gap >10 cM
	(cM)	Maker	SSR	ratio (%)	
A01	180.44	1335	8	99.62	3
A02	184.77	257	6	99.4	1
A03	247.78	671	7	99.64	2
A04	140.59	386	10	99.54	1
A05	212.66	633	5	99.04	3
A06	138.70	1082	5	99.6	4
A07	213.50	437	5	99.78	1
A08	213.43	1211	5	99.85	3
A09	207.16	428	5	99.18	4
A10	185.20	891	4	99.82	1
A11	242.24	529	6	99.17	6
A12	228.59	452	7	99.5	4
A13	183.63	517	6	99.7	3
D01	166.89	974	8	99.37	1
D02	137.87	583	8	99.45	0
D03	131.38	228	2	98.5	0
D04	119.43	446	4	98.71	1
D05	159.35	197	5	98.85	3
D06	140.40	791	6	98.22	3
D07	186.55	350	6	99.81	1
D08	164.14	542	5	98.95	3
D09	172.48	604	3	96.47	3
D10	204.98	621	10	99.81	5
D11	159.66	382	4	95.97	2
D12	206.11	780	7	97.46	3
D13	137.66	591	6	98.71	3
Total	4,665.59	15,918	153	2,574.12	64

Collinearity analysis indicating that most of markers on the genetic map were in accordance with the physical map (Figure 2B). The density of markers in the physical and genetic map indicated the high-coverage and uneven distribution of markers throughout the whole genome (Figures 2A,C).

### Analysis of Segregation Distortion Regions

A total of 80 SDRs distributed on 26 chromosomes were detected, and only five of these SDRs (locating on chromosome A01, A08, A09, D08 and D10) favored TX-832 alleles, whereas the remaining favored CCRI35 alleles (Supplementary Figure 2 and Supplementary Table 6). Remarkably, the whole chromosome of A10 (SDRA10.1) and D02 (SDRD02.1), and 95.03% of chromosome A03 (SDRA03.1) were significantly skewed toward CCRI35. In addition, the interval of other SDRs that skewed toward CCRI35 ranged from 1.16 to 114.94 cM, and their chromosome coverage ranged from 0.54 to 61.61%. As for SDRs skewed toward TX-832, their interval ranged from 5.40 to 16.19 cM, and their chromosome coverage ranged from 2.63 to 8.73%. This result suggests that fragments from the cultivar CCRI35 are more predominant than the race accession TX-832.

### Quantitative Trait Loci Identification for Fiber Quality and Yield-Related Traits

Totally, 61 FL QTLs, 62 FS QTLs, 12 FM QTLs, 50 FU QTLs, and 25 FE QTLs were identified based on six environments (Supplementary Table 7). Among them, 96 QTLs were located in the At subgenome and 114 QTLs were located in the Dt subgenome. The additive effect of five FL QTLs, three FS QTLs, seven FM QTLs, and five FE QTLs were conferred by TX-832, while the other fiber quality QTLs were contributed by CCRI35. Twenty-three FL QTLs, 12 FS QTLs, two FM QTLs, 13 FU QTLs, and two FE QTLs were detected in at least three environments. Except for *qFM_D12.1*, the additive effect origin of the other stable fiber quality QTLs were all derived from CCRI35.

A total of 73 QTLs (43 on the At subgenome and 30 on the Dt subgenome) for yield-related traits were identified, including 37 QTLs for BW, 15 QTLs for SI, 12 QTLs for LP, and 9 QTLs for LI (Supplementary Table 8). Three BW QTLs and three LP QTLs were detected in three or more environments, and four LI QTLs were identified in two environments. Especially, *qLP_D03.1* were detected in all the tested environments. The favorable alleles of all these stable yield-related QTLs were contributed by CCRI35.

**FIGURE 3:**
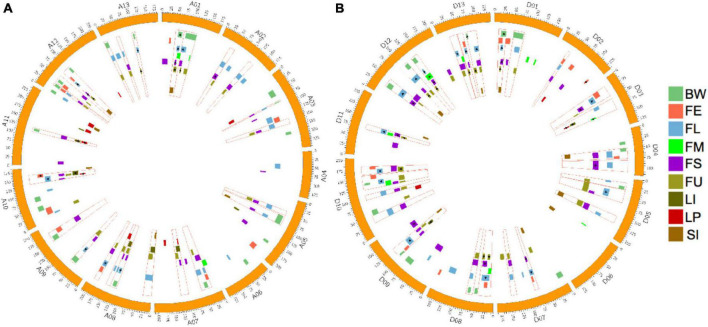
Distribution of QTLs for fiber quality and yield related traits and QTL clusters in the genetic map. **(A)** Distribution of QTLs and QTL clusters in the At subgenome. **(B)** Distribution of QTLs and QTL clusters in the Dt subgenome. The red rectangular frame indicating QTL clusters. The highlight rectangle indicating the confidence interval of each QTL, and the triangle indicating that the QTL is stable across environments.

The distribution of all identified QTLs are shown in Figure 3. To summarize, QTLs unevenly distributed on 26 chromosomes, with Chr.D10 harbored the maximum QTL (20 QTLs), followed by Chr.A08 and Chr.A12 (18 QTLs), while Chr.A04, Chr.A06, and Chr.D06 possessed only two QTLs, respectively.

### Quantitative Trait Loci Clusters Analysis

In this work, regions that contained three and more overlapped QTLs were determined to be QTL clusters. As a result, 24 QTL clusters in the At subgenome and 26 QTL clusters in the Dt subgenome were identified (Figure 3 and Supplementary Table 9). The number of clusters distributed on chromosomes ranged from one to four, while no cluster was located on Chr.A04, Chr.A06, and Chr.D06. The number of QTLs located in each cluster ranged from three to seven. A total of 213 QTLs, accounting for 75.5% of all QTLs, were contained in clusters. Twenty-seven QTL clusters, with 10 located on the At subgenome and 17 located on the Dt subgenome, contained at least one stable QTL. Totally, 59 stable QTLs were contained in QTL clusters. More detailed information about QTL clusters are summarized in Figure 3 and Supplementary Table 9.

### Validation of Major Quantitative Trait Loci Clusters

**FIGURE 4:**
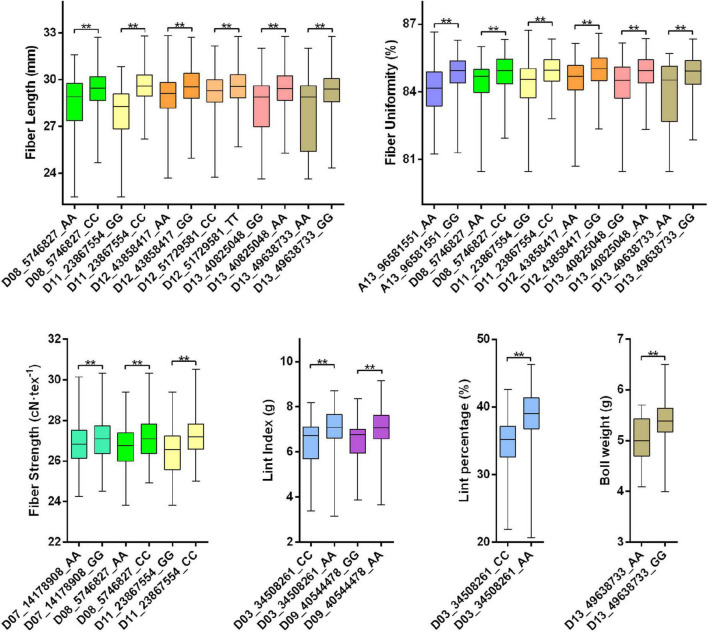
Validation of SNP markers in part of major QTL clusters.

Clusters that contained QTLs, of which PVE values larger than 10 and LOD values larger than five, were determined as major QTL clusters in this study. Totally 23 major QTL clusters were identified and analyzed (Supplementary Table 10). Eight major QTL clusters were located in the At subgenome and the other 15 were located in the Dt subgenome. SNP markers in these major QTL clusters that shared the same genome position with published SNPs data from the study of Ma Z. et al. (2018) were chosen to validate their association with target traits from 419 *G. hirsutum* accessions. Validation results of each cluster are displayed in Supplementary Table 10 and Figure 4. As a result, common SNPs are significantly associated with one or more target traits. Specially, SNPs that are located in the stable QTL regions showed extremely significant difference between alleles (Figure 4). For example, two environment-stable QTLs for yield-related traits (*qLP_D03.1* and *qLI_D03.1*) were contained in Cluster_D03.2, and accessions with different SNP (D03_34508261) alleles showed distinctively phenotype performance of these two traits. Besides, in the Cluster_D11.1, which contained three stable QTL for fiber quality (*qFL_D11.1*, *qFS_D11.2*, and *qFU_D11.1*), different SNP (D11_23867554) alleles also performed distinctively phenotype in fiber length, fiber strength, and fiber uniformity. The validation result indicates that these major QTL clusters are important phenotype selection regions during cotton domestication, and some of them accounted for large percentage of the phenotypic variation.

### Prediction of Candidate Genes in Stable Quantitative Trait Loci

In this study, four stable QTLs, with relatively narrow confidence interval, related to fiber length (*qFL_A08.2*, *qFL_A12.3*), micronaire (*qFM_D03.1*), and strength (*qFS_D08.2*) were carried out candidate gene prediction analysis. The confidence intervals of these stable QTLs were aligned to the physical genome to identify the corresponding genes. The public released RNA-seq data were analyzed. The expression levels of cell elongation stage (0–15 DPA) were focused for fiber length and secondary wall synthesis stage (15–25 DPA) were focused for fiber strength and micronaire, respectively. Together with the genome resequencing data of TX-832 and CCRI35 and the functional annotation of genes, candidate genes for the above stable QTLs were finally identified.

*qFL_A08.2* was positioned at 103.7–104.6 Mb on chromosome A08, where 35 genes were located, and 18 of them were expressed in developing fibers. Among the expressed genes, only *GH_A08G1613*, encoding a Leucine-rich repeat protein kinase family protein (LRR-RLK), showed non-synonymous variations (a frameshift deletion and two single nucleotide variations) between TX-832 and CCRI35.

*qFL_A12.3* was mapped to 77.9–79.4 Mb on chromosome A12, where 56 genes were located in this region with 30 of them expressed in the early stage of developing fibers. Eleven out of the 30 expressed genes harbored non-synonymous sequence variations between TX-832 and CCRI35. Among them, *GH_A12G1155* (*TGD4*), which participates lipid transport process, was terminated early at the 153rd amino acid in TX-832.

For the *qFM_D03.1*, it was located in at an ∼1.6 Mb region with 63 annotated genes and 23 genes expressed in the secondary wall synthesis stage. Non-synonymous sequence variations were identified in two genes that participated in activities and processes including oxidoreductase activity (*GH_D03G1329*) and microtubule-based process (*GH_D03G1338*).

*qFS_D08.2* was positioned at 5.2–5.7 Mb on chromosome D08, where 27 out of the total 47 genes were expressed in fibers of secondary wall synthesis stage. Among the 27 genes, eight genes harbored non-synonymous sequence variations between the two parents, and a gene that was involved in the brassinosteroid mediated signaling pathway (*GH_D08G0503*/*BSL1*) was identified.

## Discussion

### *Gossypium hirsutum* Race *palmeri* Accessions Contribute to Broaden the Genetic Diversity and Improve Fiber Micronaire of Cultivars

In this work, a total of 73 yield-related QTLs (10 stable QTLs) and 210 fiber quality QTLs (52 stable QTLs) were identified across six environments. In a previous work, Liu et al. (2017) established a *G. hirsutum* intraspecific RIL population and detected only 32 yield-related QTLs and 73 QTLs across six environments. Similarly, Jamshed et al. (2016) identified 165 fiber quality QTLs (47 stable QTLs) across 11 environments also using a *G. hirsutum* intraspecific RIL population. Overall, the number of total QTLs and stable QTLs in this work were more than previous analogous studies. Modern *G. hirsutum* cultivars had been domesticated in many traits including the grown habit, high yield, and superior fiber quality (Fang L. et al., 2017), causing narrow genetic basis among cultivars but rich genetic diversity between races and cultivars (Lacape et al., 2006). Besides, the difference in trait performance between mapping parents is also a causing reason of total QTL numbers. In this work, CCRI35 performed much better than TX-832 in almost all traits, except for fiber micronaire and fiber elongation. The great genetic diversity and significantly different phenotype between CCRI35 and TX-832 resulted in large amount of QTL alleles detected in this work. Therefore, *G. hirsutum* race accessions, which are both genetically and phenotypically diverse, could be utilized as resources to broaden the genetic diversity of cultivars, and further contributed to identify more favorable alleles related to important agronomic traits.

As an index to reflect the fitness and maturity of cotton fiber, fiber micronaire is one of the most significant characteristics of the textile industry. However, the complexity of fiber micronaire trait hindered its improvement in cotton breeding. On the one hand, fiber micronaire tends to variate widely across environments, and thus was excluded from many studies that focus on improving fiber quality (Wang B. et al., 2017; Ma J. et al., 2018; He et al., 2021). Compared with other fiber quality traits such as length and strength, the opportunity of detecting favorable alleles for fiber micronaire was decreased. On the other hand, the *G. hirsutum* cultivars have experienced a long period of domestication for high yield related traits, such as high lint percentage, which usually resulted in coarse fibers with high micronaire values (Song et al., 2021). In this work, the race accession TX-832 performed better than the cultivar CCRI35 in fiber micronaire in all environments. Different from other fiber quality traits, that most of their favorable alleles conferred by the cultivar CCRI35, five out of 12 FM QTLs were favorable in the race accession TX-832. TX-832 provides valuable genomic resources for the improvement of fiber micronaire. Among these QTLs, *FM_D03.1* were identified in five environments and explained 10.2–33.8% of the phenotypic variation. This QTL allele from *palmeri* accession could be preferentially utilized to improve fiber micronaire of cultivars.

### Major Quantitative Trait Loci Clusters and Common Quantitative Trait Loci Reveal Footprints of Cotton Domestication

In this work, 23 major QTL clusters were detected, and favorable alleles of QTLs (except for *qFM_D03.1*) in these clusters were all contributed by the cultivar parent CCRI35. This result suggests that these clusters are major domesticated regions during the evolution from *G. hirsutum* races to modern *G. hirsutum* cultivars. Eight major QTL clusters were located at the At subgenome whereas the other 15 were located in the Dt subgenome, suggesting that the Dt subgenome might have experienced stronger domestication and selection than the At subgenome, which was consistent with previous studies (Wang M. et al., 2017; Ma Z. et al., 2018). Associated studies of common SNPs in these regions verified major QTL clusters, but also suggested that unfavorable allele still exist in part of modern cultivars. These SNP markers could be applied as an effective molecular tool to select varieties with high yield and superior fiber quality in future cotton breeding.

A total of 72 stable QTLs were identified, and many of them had been reported in previous studies (Shen et al., 2005, 2019; Zhang et al., 2011; Su et al., 2013; Yu et al., 2013; Shang et al., 2015, 2016; Guo et al., 2018; Deng et al., 2019; Ma et al., 2019; Chen et al., 2020). By comparing the physical confidence interval of QTLs in different studies, 35 stable QTLs in this work were found sharing overlapped interval with previous reported QTL (Supplementary Table 11), and 15 of them were detected in more than one previous work. Among them, *qFL_A10.3* was found to be the same with QTLs in other five works (Liang et al., 2013; Shao et al., 2014; Wang et al., 2015; Wang H. et al., 2016; Shi et al., 2019), and *qFL_D11.1* and *qLP_D03.1* were found to be the same with QTLs in other four works, respectively (Wang et al., 2013, 2020; Ning et al., 2014; Shi et al., 2015; Jamshed et al., 2016; Diouf et al., 2018; Ma J. et al., 2018). Six QTLs (*qFE_D02.3*, *qFL_A08.5*, *qFL_D07.1*, *qFL_D13.3*, *qFS_D01.1*, *qFS_D07.1*) were identified in other three previous works (Shen et al., 2006; Feng et al., 2015; Tang et al., 2015; Wang F. et al., 2016; Zhang et al., 2016). These common QTLs identified across different populations and environments suggest that they are important domesticated alleles with stable genetic effects, and will provide valuable reference for future marker-assisted breeding of cotton varieties with high-yield and eminent fiber quality (Shi et al., 2020).

### Candidate Genes for Stable Quantitative Trait Loci

In this work, we focused on four stable QTLs for fiber length, strength, and micronaire, and five candidate genes were identified. In the confidence interval of *qFL_A08.2*, only *GH_A08G1613*, which encodes a leucine-rich repeat protein kinase family protein (LRR-RLK), was detected as the candidate gene. A few *LRR-RLK* genes have been suggested to be functional during fiber development in cotton researches (Islam et al., 2016; Fang X. et al., 2017; Sun et al., 2018). LRR–RLKs were previously reported to be involved in pathways that regulate cellulose deposition (Xu S. et al., 2008) and cell expansion (Dai et al., 2013). The regulation of *GH_A08G1613* on fiber length might also occur through these two pathways. *GH_A12G1155* was identified for *qFL_A12.3*, and it is a homolog of *AT2G44640* (TRIGALACTOSYLDIACYLGLYCEROL-like protein, *TGD4*) in Arabidopsis. *AtTGD4* was reported to be required for lipid transport (Xu C. et al., 2008). It has been demonstrated that during fiber elongation, continuous synthesis and transport of lipids and proteins are crucial for supporting the enlargement of vacuoles and plasma membrane (Wanjie et al., 2005). The length of fiber was significantly reduced in the lipid transfer protein *GhLTPG1*-knockdown cotton plants (Deng et al., 2016). In the accession TX-832, a single nucleotide variation resulted in the early termination of *GH_A12G1155* protein, suggesting that it might affect the lipid transport pathway and further influence fiber length.

For *qFM_D03.1*, two genes were identified with non-synonymous sequence variations between TX-832 and CCRI35, including the probable NAD(P)H dehydrogenase (quinone) FQR1-like 3 (*GH_D03G1329*) and tubulin beta chain 3 (*GH_D03G1338*). NAD(P)H dehydrogenase was involved in the oxidoreductase activity that was associated with reactive oxygen species (ROS) metabolism, and ROS levels were known to play vital roles in fiber elongation and secondary cell wall synthesis (Wang M. et al., 2017). Tubulin proteins are the major structural components of microtubules which undergo several phases of reorganization during cotton fiber development (Seagull, 1992). Microtubules are core elements of the cytoskeleton and are closely related to cellulose deposition and secondary cell wall development in fibers (Deng et al., 2016; Chen et al., 2021).

Phytohormones play vital roles in regulating cotton fiber development (Xiao et al., 2019). Brassinosteroid (BR) signaling has been reported to affect the deposition of cellulose into secondary cell wall of cotton fibers, and increasing the expression of the a brassinosteroid receptor BRI1 produced cotton fibers with thicker secondary cell walls (Sun et al., 2015). *GH_D08G0503* in the interval of *qFS_D08.2* was annotated to encode a BR1 SUPPRESSOR 1 (*BSU1*)-like protein (*BSL1*). *BSU1* are required for signal transduction from *BR1* to downstream proteins (Kim et al., 2011). Therefore, *BSL1* might play the same role as *BSU1*, and it affects fiber strength through participating in the BR signal pathway and further regulating the deposition of cellulose into secondary cell wall.

Based on the above analysis, five candidate genes related to fiber quality were identified. But further functional analysis, such as generating genetic modified cotton plants, is needed to be conducted to verify these candidate genes.

## Data Availability Statement

The datasets presented in this study can be found in online repositories. The names of the repository/repositories and accession number(s) can be found below: https://www.ncbi.nlm.nih.gov/, PRJNA781434.

## Author Contributions

ZZ designed and supervised the research. XL and LY performed the data analysis and wrote the manuscript. JW, ZG, ZT, and DJL established the population and participated in the field experiment. YQW, QL, and JY extracted the DNA for sequencing library construction. YLW, LC, DXL, and KG collected the phenotype data. All authors have read and approved the manuscript.

## Conflict of Interest

The authors declare that the research was conducted in the absence of any commercial or financial relationships that could be construed as a potential conflict of interest.

## Publisher’s Note

All claims expressed in this article are solely those of the authors and do not necessarily represent those of their affiliated organizations, or those of the publisher, the editors and the reviewers. Any product that may be evaluated in this article, or claim that may be made by its manufacturer, is not guaranteed or endorsed by the publisher.

## Footnotes

1
http://cotton.zju.edu.cn/
