# Supplementary figures and images for: Analyzing Quantitative Trait Loci for Fiber Quality and Yield-Related Traits From a Recombinant Inbred Line Population With Gossypium hirsutum Race palmeri as One Parent
Source: Front Plant Sci. 2022 Jan 3;12:817748. doi: 10.3389/fpls.2021.817748 (PMC8763314; doi:10.3389/fpls.2021.817748)

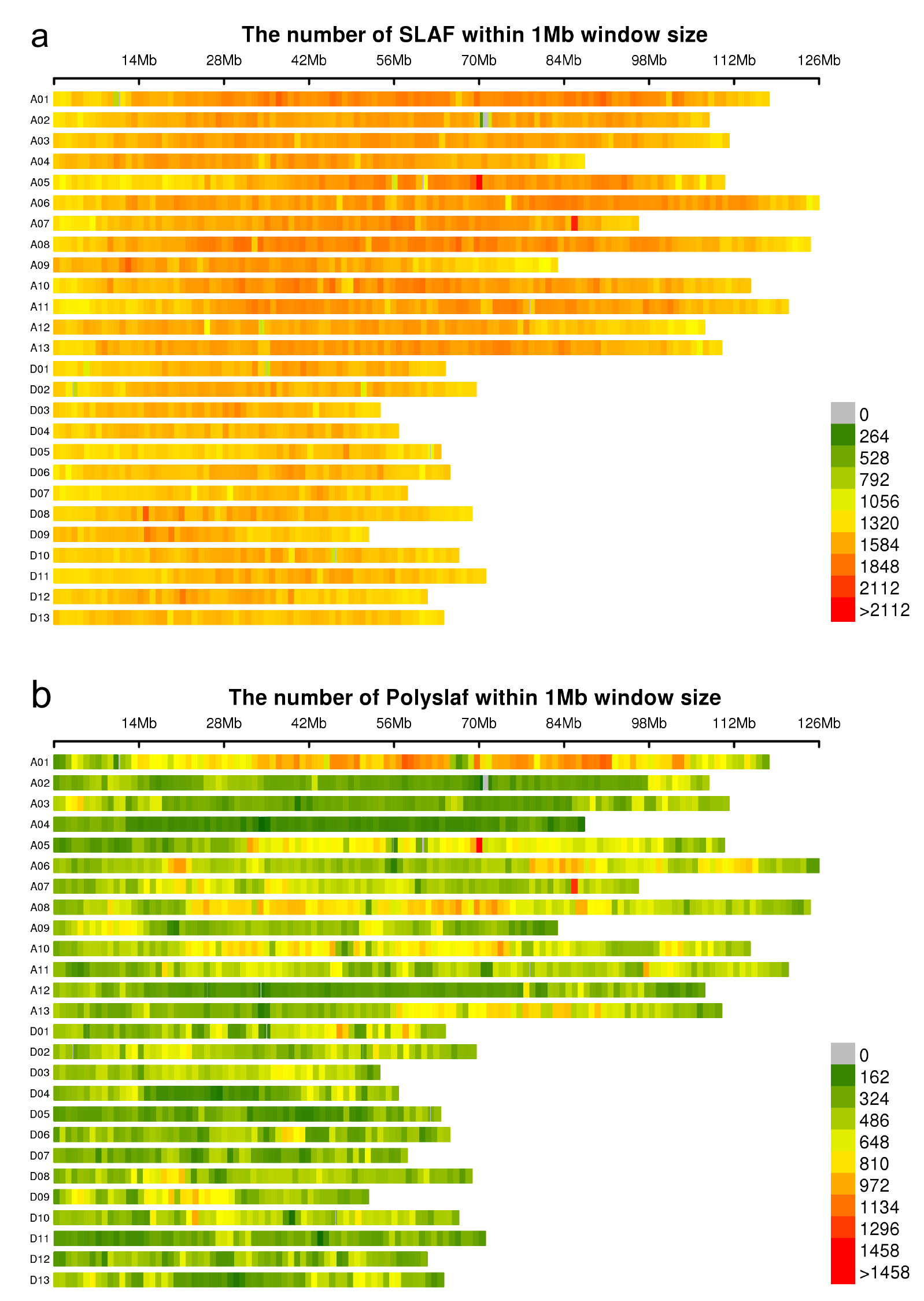

Supplement: Supplementary file 12 [file Image_1.TIF]

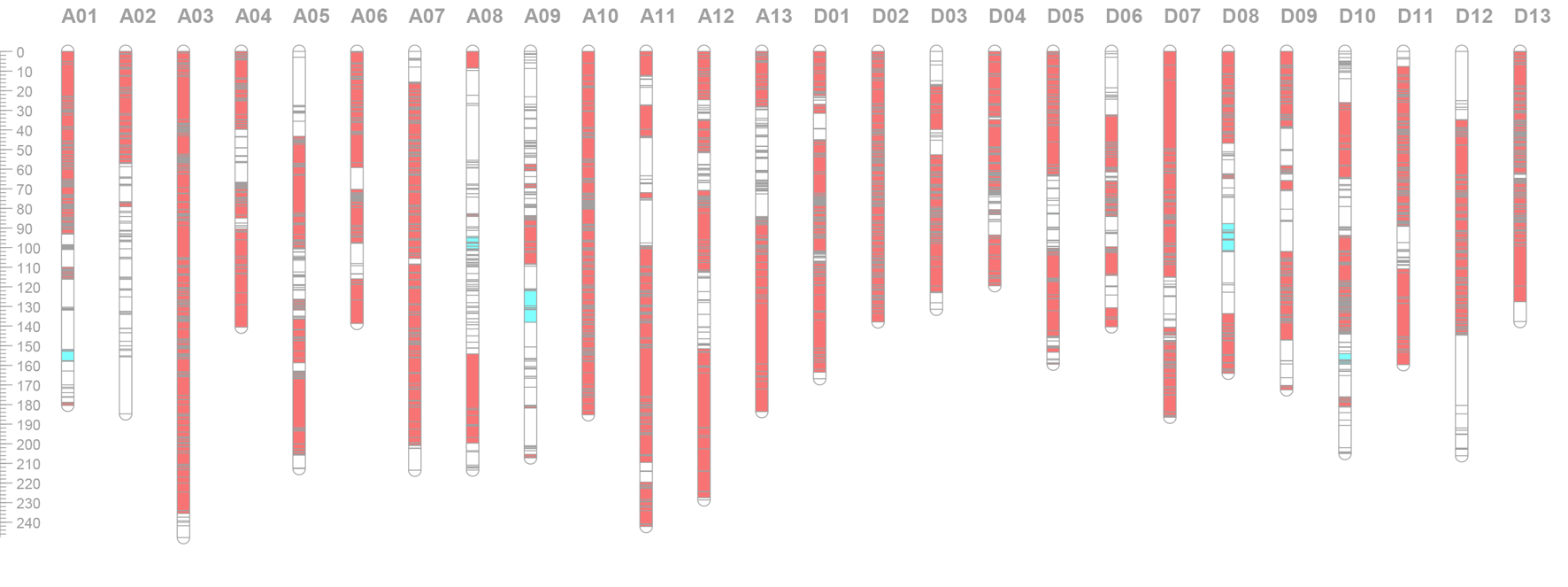

Supplement: Supplementary file 13 [file Image_2.TIF]
